# Amphiregulin mediates progesterone-induced mammary ductal development during puberty

**DOI:** 10.1186/bcr3431

**Published:** 2013-05-25

**Authors:** Mark D Aupperlee, Jeffrey R Leipprandt, Jessica M Bennett, Richard C Schwartz, Sandra Z Haslam

**Affiliations:** 1 Department of Physiology, Michigan State University, 567 Wilson Rd., East Lansing, MI 48824, USA; 2 Department of Microbiology and Molecular Genetics, Michigan State University, 567 Wilson Rd., East Lansing, MI 48824, USA

## Abstract

**Introduction:**

Puberty is a period of increased susceptibility to factors that cause increased breast cancer risk in adulthood. Mammary end buds (EBs) that develop during puberty are believed to be the targets of breast cancer initiation. Whereas the role of estrogen (E) has been extensively studied in pubertal mammary gland development, the role of progesterone (P) during puberty is less defined.

**Methods:**

Pubertal and prepubertal ovariectomized mice were treated with vehicle control (C), E, P, or E+P. Mammary glands from these mice were analyzed for changes in morphology, proliferation, and expression of the downstream targets amphiregulin (AREG) and receptor activator of NF-κB ligand (RANKL).

**Results:**

P, acting specifically through the progesterone receptor, induced increases in mammary gland proliferation and EB formation that were associated with increased AREG expression in ducts and EBs. E, acting specifically through the estrogen receptor, produced similar responses also mediated by AREG. Blocking AREG action by treatment with an EGFR inhibitor completely abrogated the effect of P on EB formation and proliferation and significantly reduced proliferation within ducts. P also increased expression of RANKL, primarily in ducts. Treatment with RANK-Fc, an inhibitor of RANKL, reduced P-dependent proliferation in ducts and to a lesser extent in EB, but did not cause EB regression.

**Conclusions:**

These results demonstrate a novel P-specific effect through AREG to cause EB formation and proliferation in the developing mammary gland both before and during puberty. Thus, hormones and/or factors in addition to E that upregulate AREG can promote mammary gland development and have the potential to affect breast cancer risk associated with pubertal mammary gland development.

## Introduction

The mouse mammary gland is used as a model for development of the human breast. Before puberty, both the mouse and human mammary glands grow and develop at an isometric rate, at the same rate as the body. At puberty, rapid changes in the hormonal milieu drive increased proliferation and expanded ductal development to fill the fat pad (reviewed in [1]). During this time, highly proliferative structures called end buds (EBs) are present at the leading growth front of ducts in the gland (reviewed in [2]). EBs progress into the mammary fat pad, and the ductal network is formed by bifurcation of the EBs and branching of ducts until the fat pad is filled with an extensive, branched ductal system. As the mammary gland reaches the limits of the fat pad, these EB structures regress and disappear.

There is increasing interest in factors influencing pubertal mammary gland development and breast cancer risk later in life [3]. EB structures are sensitive to chemical carcinogens [4,5], and compounds that influence EB formation could alter the sensitivity of the mammary gland to chemical carcinogen action. In mouse and rat models of mammary carcinogenesis, the peripubertal period shows increased sensitivity to carcinogen exposure and mammary cancer development [6-8]. During adolescence, maturation of the hypothalamic-pituitary-ovarian axis, the regulator of ovarian hormone production, occurs along with the majority of breast development. Of particular interest are factors, such as endocrine disruptors, that may influence ovarian hormone production or hormone action in the developing mammary gland. To understand factors that influence hormone action, it is important to first have an understanding of the mechanisms of normal hormone responses in the mammary gland.

The ovarian hormone estrogen (E) is the primary hormone required for ductal development during puberty [9], whereas the role of progesterone (P) during puberty is less defined. P acting through the progesterone receptor (PR) is not essential for pubertal mouse mammary gland growth, as the PR knockout (PRKO) mouse completes ductal development successfully [10]. Despite this, a role for P and PR during puberty is indicated by a delay in ductal development in mice treated with the PR-antagonist RU486 and in the PR-knockout (PRKO) mouse [11]. In addition, treatment of IGF-I^-/- ^mice with IGF-I plus P is capable of stimulating mammary gland development in the absence of E, suggesting that P has E-independent effects in the pubertal mammary gland [12]. A role for P in increasing ductal branching during puberty has also been demonstrated [13-15]. Consistent with pubertal P being important for both normal development and breast cancer development, supplementation of carcinogen-treated pubertal rats with E + P results in higher tumor incidence than does treatment with E alone [16]. Thus, P and PR can play an important role in pubertal mammary gland development, and may also be involved in increased cancer susceptibility during puberty. However, no mechanism for P action in the pubertal mammary gland has been described.

In this study, we sought to elucidate further the role of P in pubertal ductal development. We found that P acting through PR caused pubertal EB formation, which was dependent on an increase in amphiregulin (AREG) expression. P-induced AREG was associated with proliferation in both the EBs and ducts. P also increased expression of receptor activator of NF-κB ligand (RANKL), a known paracrine mediator of P, which primarily affected proliferation in ducts. Further showing the importance of AREG downstream of P, we found that the prepubertal mammary gland was highly sensitive to low doses of P that also increased AREG expression and led to EB formation. These studies further emphasize the importance of AREG for EB formation in the pubertal gland and demonstrate that hormones and/or factors, in addition to E, may be important for the early regulation of AREG.

## Materials and methods

### Mice

BALB/c and C57BL/6 mice were purchased from Harlan (Indianapolis, IN, USA) and Jackson Laboratory (Bar Harbor, ME, USA), respectively. Two time frames of mammary gland development were examined. In the first case, pubertal 4-week-old BALB/c and C57BL/6 mice that initiated estrus cycling and exhibited EBs were ovariectomized (OVX). Three weeks post-OVX recovery was allowed for complete EB regression before hormone treatments [17]. OVX mice were injected daily for 5 days with saline control (C), 17-β-estradiol (E2) (1 μg/inj), progesterone (P) (1 mg/inj), or E2+P (1 μg + 1 mg/inj, respectively). These concentrations of E2 and P have been used in previous studies to examine the effects of E2 and P in the adult mammary gland and were used to compare pubertal versus adult sensitivity and responses to the hormones [18]. To block PR-mediated effects, anti-progestin RU486 (1.3 mg/inj) (mifepristone; Sigma, St. Louis, MO, USA) was co-injected with P. To block estrogen receptor (ER)-mediated effects, anti-estrogen ICI 182,780 (ICI) (1.1 μg/inj) (Tocris Bioscience, Ellisville, MO, USA) was co-injected with E2. To block epidermal growth factor receptor (EGFR)-mediated effects, E2- and P-treated mice were given gefitinib (Iressa; ChemieTek, Indianapolis, IN, USA) (300 mg/kg dissolved in corn oil) daily for 5 days by oral gavage. To block RANKL-mediated effects, P-treated mice were co-injected with RANK-Fc (20 mg/kg dissolved in saline) (R&D Systems, Minneapolis, MN, USA) every other day for 5 days.

In the second case, prepubertal 3-week-old BALB/c mice that had not started estrus cycles or EB formation were OVX. Two weeks post-OVX mice received a single injection with saline control (C), or low-dose E2 (0.1 μg), P (0.1 mg), or E2+P (0.1 μg E + 0.1 mg P) with or without 5 mg ICI 182,780 (ICI) (Tocris Bioscience), and then killed 48 hours later. Hormone dosages were based on the minimal concentrations of E2 capable of EB formation in the pubertal gland [19]. To block local PR-mediated effects in the mammary gland, elvax pellets containing the anti-progestin RU486 (1 μg mifepristone; Sigma) were implanted into the right number 4 mammary gland of prepubertal OVX mice 2 weeks after OVX. Control pellets were implanted into the left number 4 mammary gland. The following day, mice were given a single injection of P (0.1 mg).

All mice were injected with 5-bromo-2'-deoxyuridine (BrdU) (70 μg/g body weight) 2 hours before being killed. Mammary glands were fixed and processed as whole mounts [20], or paraffin-embedded for immunohistochemistry [21]. All animal experimentation was conducted in accord with accepted standards of humane animal care and approved by the All University Committee on Animal Use and Care at Michigan State University.

### Immunofluorescence

PR was detected by using mouse monoclonal anti-PR (1:50; hPRa7; Neomarkers, Fremont, CA, USA). Estrogen receptor α (ERα), AREG, RANKL, and BrdU were detected by using mouse monoclonal anti-ERα (1:10; Novocastra; Leica Microsystems Inc., Buffalo Grove, IL, USA), goat polyclonal anti-amphiregulin (1:100; R&D Systems, Minneapolis, MN, USA), goat polyclonal anti-RANKL (1:500; R&D Systems), or mouse monoclonal anti-BrdU (undiluted; kit from Amersham Biosciences, Piscataway, NJ, USA) primary antibodies followed by appropriate secondary antibodies conjugated to Alexa 488 (Molecular Probes, Eugene, OR, USA) [21]. Double labeling of PR or ERα with anti-BrdU antibody was as described previously [21], by using appropriate secondary antibodies conjugated to Alexa 488 or Alexa 546. Nuclei were counterstained with 4',6-diamidino-2-phenylindole, dilactate (DAPI) (Molecular Probes). Sections were visualized and images captured by using a Nikon inverted epifluorescence microscope (Mager Scientific, Dexter, MI, USA) with MetaMorph software (Molecular Devices Corporation, Downington, PA, USA).

### Quantitative RT-PCR

Whole mammary gland total RNA was extracted by using TRIzol (Invitrogen, Carlsbad, CA, USA) following the manufacturer's suggested protocol. cDNA was produced by reverse transcription with random hexamer primers and reverse transcriptase-polymerase chain reaction (RT-PCR), and quantitation of murine amphiregulin (Mm00437583_m1) (Applied Biosciences, Carlsbad, CA, USA) and 18S RNAs (Hs99999901_s1) was performed, as previously described [22].

### Quantitation and statistical analyses

BrdU and PR were quantitated for the number of positive luminal epithelial cell nuclei from captured images by using MetaMorph software, as previously described [18]. A minimum of three mice per treatment group and a minimum of 500 cells in three independent sections per mouse were analyzed for all experiments. Whole-mount preparations of mammary gland number 4 or numbers 2/3 were scored for numbers of EBs. EBs were defined as enlarged (>100 μm in diameter), multilayered ductal tips surrounded by adipocytes and located at the periphery of the gland. Results are expressed as mean ± SEM, and differences are considered significant at *P *< 0.05 with the Student *t *test or ANOVA, as appropriate.

## Results

### Morphologic and proliferative responses to hormones during puberty

**Figure 1:**
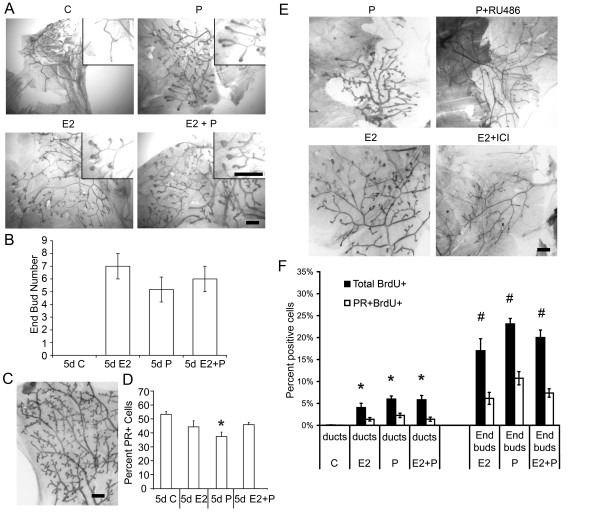
**Both 17-β-estradiol and progesterone induce similar morphologic and proliferative responses in the pubertal mammary gland**. Pubertal 4-week-old BALB/c mice were OVX, allowed to recover for 3 weeks, and then treated for 5 days with vehicle control (C), E2, P, or E2+P, as described in the Materials and Methods section. **(A) **Morphologic response to 5d C, E2, P, or E2+P. Note lack of EBs in controls and similar presence of EBs in E2-, P-, and E2+P-treated glands. Scale bar, 1 mm. **(B) **End bud (EB) quantitation. Values represent the mean ± SEM number of EBs in a number 4 mammary gland (*n *= 3 mice). **(C) **Morphologic response to 5d P in the adult 17-week-old BALB/c OVX mammary gland; note presence of extensive side branching. Scale bar, 1 mm. **(D) **Immunofluorescent detection of PR expression in luminal cells. The values represent the mean ± SEM percentage PR-positive cells (*n *= 3 animals per treatment). The percentage PR-positive cells in 5-day P-treated BALB/c mice was less than control (*P < 0.05). **(E) **Inhibition of EB formation by E2+ ICI 182,780 and P+RU486. Scale bar, 1 mm. **(F) **Proliferation analysis by dual immunofluorescent detection of BrdU and PR. The total percentages BrdU-positive cells and BrdU-positive cells co-expressing PR in ducts and EBs are presented. The values represent the mean ± SEM (*n *= 3 animals per treatment). The percentage of BrdU-positive luminal epithelial cells in ducts after E2, P, or E2+P treatment was greater than control (*P < 0.05). The percentage of BrdU-positive cells was greater in EBs than in ducts after E2, P, or E2+P treatment (#*P *< 0.05).

Before puberty and the onset of estrus cycles, mammary ductal outgrowth is limited and hormone independent. Increasing hormone levels during puberty produce localized mammary ductal proliferation at sites called end buds (EBs) that drive ductal elongation (reviewed in [23]). To examine the role of progesterone (P) during puberty, 4-week-old mice were OVX to remove endogenous hormone production and allow EB regression, and then hormone-treated for 5 days. Two mouse strains (BALB/c, C57BL/6) were used in these studies because they showed differential sensitivity of the adult gland to P [24]. Thus, it was of interest to determine whether the differential P sensitivity also occurred in the pubertal gland. Treatment of BALB/c mice for 5 days with P alone stimulated EB formation (Figure 1A). The degree of stimulation (EB formation) obtained with P alone was similar to that obtained with 17-β-estradiol (E2) alone, or with E2 + P (Figure 1B). EBs were completely regressed and absent in vehicle-treated controls. Limited sidebranching was observed in response to P or E2+P in the pubertal mammary gland in contrast to the extensive sidebranching normally seen in the adult BALB/c mammary gland at the same hormone doses [24] (Figure 1C). EB formation with P treatment was also obtained in C57BL/6 pubertal mice (see Additional File 1, Figure S1A). The similarity of the pubertal hormone responses, particularly for P, between the BALB/c and C57BL/6 mice was in contrast to the significant differences in mammary gland responses previously reported for P treatment of adult mice [24].

Because P exerts its effects in the mammary gland through the PR, PR protein expression and regulation by hormone treatment in pubertal BALB/c mice was examined with immunofluorescence (Figure 1D). P treatment significantly decreased the percentage of PR positive (PR^+^) luminal epithelial cells compared with vehicle controls, whereas treatment with E2 or E2+P did not significantly change the percentage of PR^+ ^cells. Hormonal regulation of PR expression was similar in both BALB/c and C57BL/6 mice (see Additional File 1, Figure S1B).

To distinguish between the mechanisms of P and E action in pubertal EB formation, anti-estrogen and anti-progestin were used to block E2 and P action through their cognate receptors, ERα and PR. Anti-progestin RU486 treatment inhibited P-induced EB formation and treatment with the anti-estrogen ICI 182,780 inhibited E2-induced EB formation (Figure 1E). Thus, the observed effects of P on EB formation in the pubertal mouse mammary gland were P-specific and mediated through PR.

### Proliferative response to hormones during puberty

The majority of proliferating cells in the adult mammary gland are ER/PR negative [18,25], suggesting a paracrine mechanism for hormone-induced proliferation. To determine whether a similar paracrine mechanism for P-induced proliferation occurred in the pubertal mammary glands, DNA synthesis, as measured by BrdU uptake into hormone-receptor positive versus negative cells, was analyzed by examining co-localization of BrdU with PR expression (Figure 1F).

The percentage of BrdU-positive (BrdU^+^) cells was significantly increased throughout the mammary gland in both ducts and EBs by E2, P, and E2+P treatment (Figure 1F). A significant percentage of PR-positive (PR^+^) cells incorporated BrdU in the pubertal mammary gland after hormone treatment. Treatment with E2, P, or E2+P produced similar BrdU uptake in PR^+ ^cells and represented about one third of the total BrdU^+ ^cells in EBs (Figure 1F). The overall percentages of BrdU^+ ^cells and BrdU uptake in PR^+ ^cells were significantly higher in EBs than in ducts. Similar results for the overall percentages of BrdU^+ ^cells and BrdU uptake in PR^+ ^cells were obtained in the C57BL/6 mammary gland (see Additional File 1, Figure S2). These BrdU-uptake results demonstrate an increased proliferative response of PR^+ ^cells to hormones in the pubertal mammary gland compared with the adult gland [18], and show a similar pattern of proliferative response in the two mouse strains.

### Amphiregulin mediates P- and E2-induced proliferation

The similarities between E2- and P-induced EB formation and proliferative responses and the lack of additional increases in proliferation with combined E2 + P, suggested a common mechanism of action for both hormones. Ciarloni *et al. *[19] previously established that E-induced amphiregulin (AREG) in mammary epithelial cells is a paracrine mediator of EB formation. AREG is the most highly expressed epidermal growth factor receptor (EGFR) ligand in ducts and end buds during puberty, and AREG is required for normal ductal morphogenesis [26]. P has also been shown to increase AREG expression in the adult mouse mammary gland [27] and mouse uterus [28]. Given the functional significance of AREG in E-induced EB formation, its high expression in the pubertal mammary gland compared with other EGFR ligands, and reported regulation of AREG by P, we hypothesized that P-induced EB formation was also mediated by AREG.

**Figure 2:**
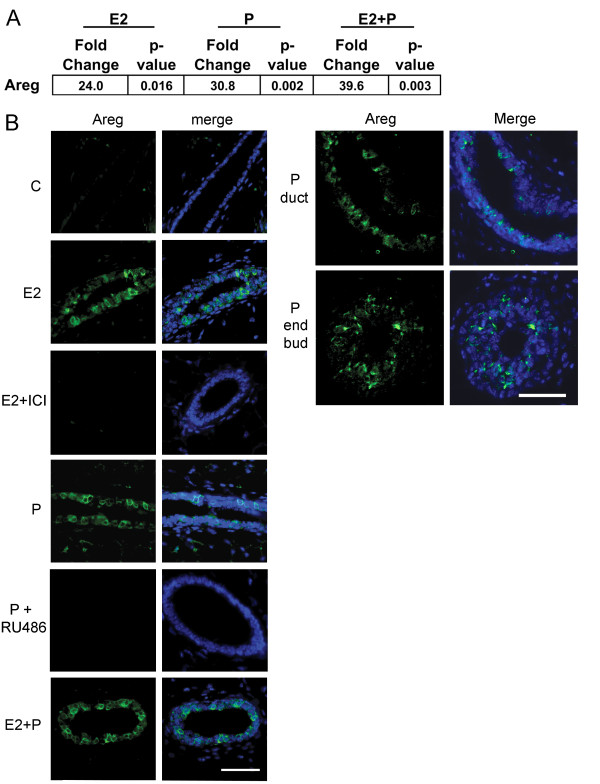
**Both 17-β-estradiol and progesterone regulate amphiregulin through their cognate receptors in the pubertal mammary gland**. Pubertal 4-week-old BALB/c mice were OVX, allowed to recover for 3 weeks, and then treated for 5 days with vehicle control (C), E2, P, or E2+P, as described in the Materials and Methods section. **(A) **RT-PCR analysis of amphiregulin (AREG) in response to hormone treatments. Fold change is relative to control treatment. **(B) **Immunofluorescent detection of AREG (green) in ducts after E2, E2+ICI, P, P+RU486, and after P treatment in both ducts and end buds. Nuclei were counterstained with DAPI (blue). Scale bar, 25 μm.

Analysis of *Areg *expression with RT-PCR in the 5-day hormone-treated pubertal mammary gland showed that *Areg *mRNA expression was significantly increased by 5-day treatment with P (30.8 fold), E2 (24.0 fold), and E2+P (39.6 fold) compared with vehicle controls (Figure 2A). AREG protein expression also increased in the pubertal mammary gland in response to 5-day treatment with E2, P, or E2+P (Figure 2B). Similar increases in AREG protein expression were also observed after hormone treatment in the C57BL/6 strain of mice (Additional File 1, Figure S3A). The E2-induced increase in AREG expression was blocked by the ERα antagonist ICI 182,780, and P-induced AREG expression was blocked by the PR antagonist RU486 (Figure 2B). Pubertal AREG expression in response to P was localized throughout the mammary gland in both ducts and EBs (Figure 2C).

**Figure 3:**
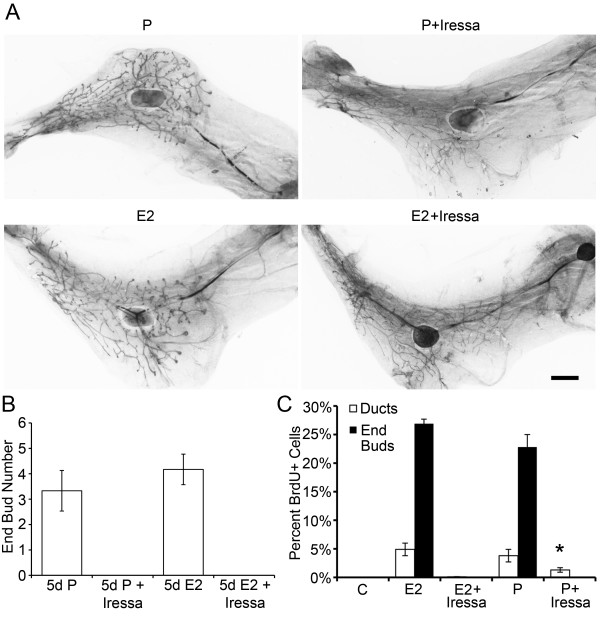
**Iressa inhibits both 17-β-estradiol and progesterone-induced proliferation and EB formation**. Pubertal 4-week-old BALB/c mice were OVX, allowed to recover for 3 weeks, and then treated for 5 days with vehicle control (C), E2 +/- Iressa (300 mg/kg), or P +/- Iressa, as described in the Materials and Methods section. **(A) **EB formation after 5 days E2, E2+Iressa, P, or P+Iressa. Note the absence of EBs in Iressa-treated mammary glands. Scale bar, 2 mm. **(B) **End bud quantitation. Values represent the mean ± SEM number of end buds in a number 4 mammary gland (*n *= 5 mice). **(C) **The percentage of proliferating cells in ducts and EBs was determined by immunofluorescence detection of BrdU incorporation. P+Iressa treatment reduced proliferation in ducts relative to P treatment (*P < 0.05).

To examine further the role of AREG in pubertal P-induced EB formation, the EGFR-inhibitor Iressa was used to block AREG action through the EGFR. Inhibition of EGFR by Iressa completely inhibited both E2- and P-induced pubertal EB formation (Figure 3A, B) and EB proliferation in the mammary gland (Figure 3C); no EBs were present in mammary gland sections from Iressa-treated mice (Figure 3A, B). The percentage of cells incorporating BrdU within ducts in response to E2 was reduced by 98% by Iressa (*P *< 0.01 versus E2), whereas the percentage of BrdU^+ ^cells in ducts in response to P was reduced 66% by Iressa (*P *< 0.05 versus P).

### RANKL in P-mediated proliferation

**Figure 4:**
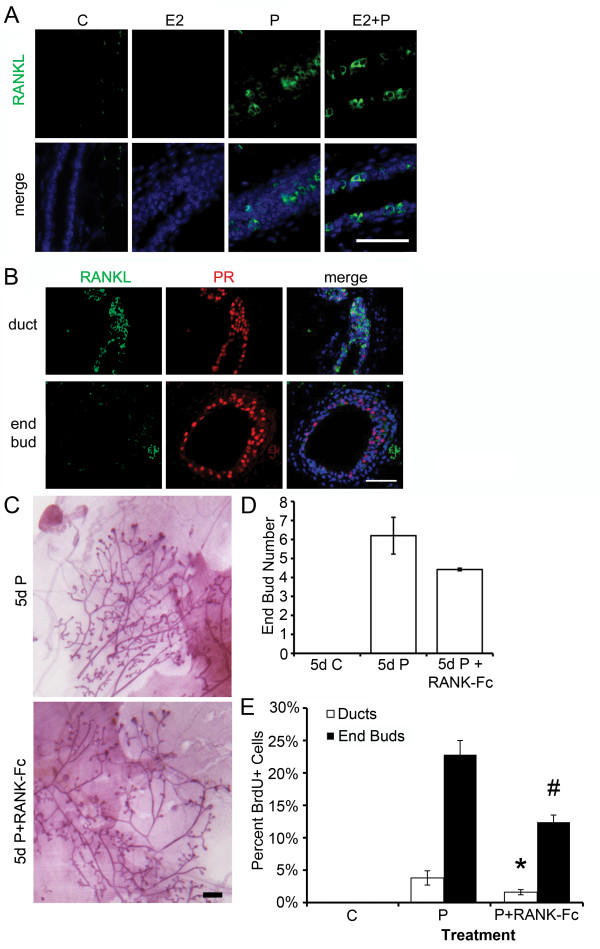
**Progesterone-induced RANKL expression contributes to the proliferative response in the pubertal mammary gland**. Pubertal 4-week-old BALB/c mice were OVX, allowed to recover for 3 weeks, and then treated for 5 days with vehicle control (C), E2, P, or E2+P, as described in the Materials and Methods section. **(A) **Immunofluorescent detection of RANKL (green) in P and E2+P-treated mammary glands. **(B) **Dual immunofluorescent detection of RANKL (green) and PR (red) expression in representative images of an E2+P-treated duct and end bud. RANKL was most strongly expressed in ducts. Nuclei **(A, B) **were counterstained with DAPI (blue). Scale bars, 25 μm. **(C) **Effect of 5d P versus 5-day P+RANK-Fc on EB formation; note lack of significant EB regression with RANK-Fc treatment. Scale bar, 1 mm. **(D) **End-bud quantitation. Values represent the mean ± SEM number of end buds in the thoracic mammary gland (*n *= 3 mice). **(E) **The percentage of proliferating cells in ducts and EBs was determined by immunofluorescent detection of BrdU incorporation after C, P, and P+RANK-Fc treatment. P+RANK-Fc treatment had fewer proliferating cells in ducts than in P treatment (*P < 0.05). P+RANK-Fc treatment had fewer proliferating cells in EB than in P treatment (#*P *< 0.05).

Receptor activator of NF-κB ligand (RANKL) is another downstream effector of P in the mammary gland [24,29]. Five-day P or E2+P treatment in the pubertal mammary gland increased RANKL expression, whereas treatment with E2 alone did not increase RANKL expression (Figure 4A). P-induced RANKL expression colocalized with PR expression (Figure 4B) and was primarily localized to ducts, and, to a lesser extent, in EBs. In contrast to BALB/c mice, 5-day P treatment of C57BL/6 mice failed to increase RANKL expression (Additional File 1, Figure S3B). However, both the pubertal BALB/c and C57BL/6 mammary glands showed expression of RANKL in response to E2+P treatment. These results indicate that P-induced RANKL is not required for EB formation and proliferation in C57BL/6 mice.

The potential role of RANKL downstream of P in EB formation and proliferation in BALB/c mice was examined by using the RANKL inhibitor RANK-Fc; RANK-Fc binds RANKL and inhibits binding to its natural receptor, receptor activator of NF-κB (RANK). Treatment with RANK-Fc had a small effect to reduce P-induced EB formation that did not achieve statistical significance (*P *= 0.2) (Figure 4C, D). In contrast, RANK-Fc significantly decreased proliferation by 58% in ducts (*P *< 0.05 versus P) and by 46% in EBs (*P *< 0.05 versus P) (Figure 4E).

### P-induced EB formation in prepubertal mice

**Figure 5:**
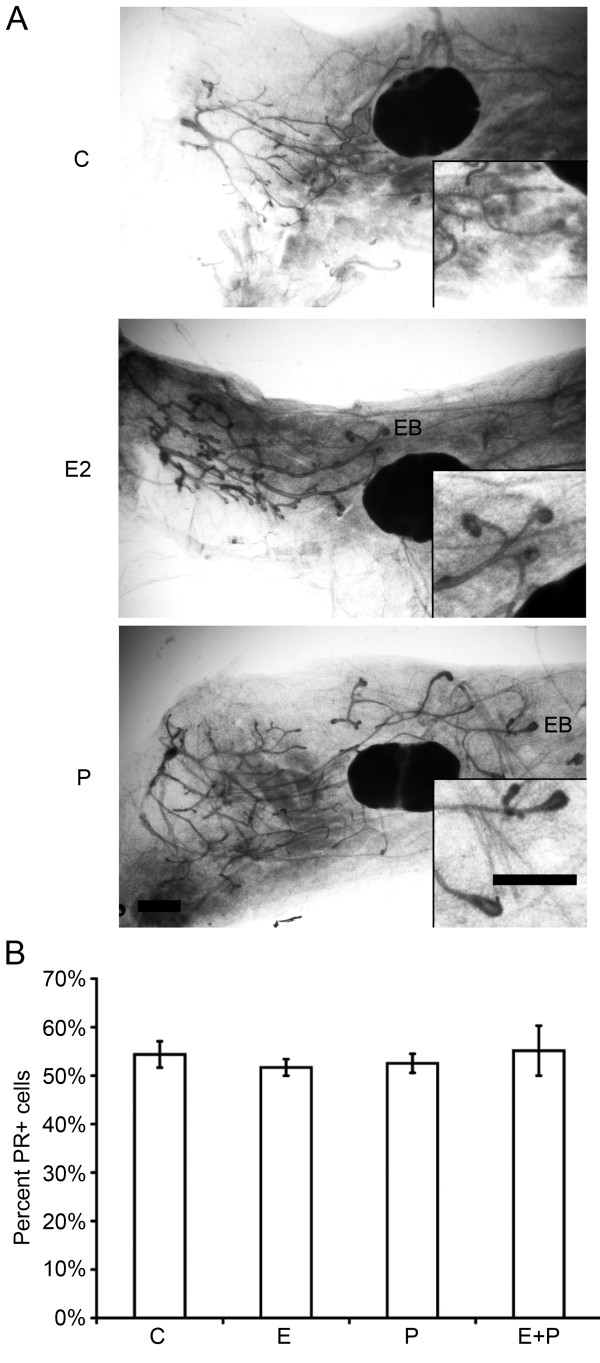
**Both 17-β-estradiol and progesterone induce morphologic responses in the prepubertal mammary gland**. Prepubertal 3-week-old BALB/c mice were OVX, allowed to recover for 2 weeks, and then treated once with vehicle control (C), E2, P, or E2+P, as described in the Materials and Methods section. **(A) **End bud (EB) formation in response to C, E2, and P. Inset shows higher magnification of EB. Note similar EB formation after E2 or P treatments. Scale bar, 1 mm. **(B) **PR expression. Immunofluorescent detection of PR-positive luminal cells. The values represent the mean ± SEM percentage of PR-positive cells (*n *= 3 animals per treatment).

**Figure 6:**
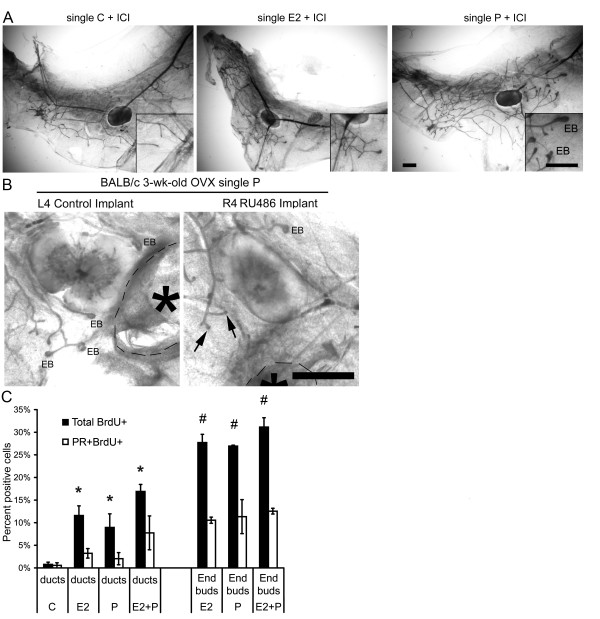
**Both 17-β-estradiol and progesterone-induced responses in the prepubertal mammary gland occur through their cognate receptors**. **(A) **Representative whole mounts from prepubertal 3-week-old BALB/c mice that were OVX, allowed to recover for 2 weeks, and then treated once with vehicle control (C) + ICI 182,780 (ICI), E2 + ICI, or P + ICI, as described in the Materials and Methods section. Scale bar, 1 mm. Inset shows higher magnification of end buds (EBs), which were present in only the P + ICI treatment group. Scale bar, 1 mm. **(B) **Effect of RU486 implant on P-induced EB formation. RU486 pellet implanted in the right mammary gland number 4, and control pellet implanted in the contralateral number 4 mammary gland. Implants are outlined with dashed lines and noted with an asterisk. Note stimulated EB in control L4 gland and nonstimulated duct ends (arrows). Scale bar, 2 mm. **(C) **Proliferation analysis by dual immunofluorescence detection of BrdU and PR. Total percentage BrdU-positive cells and percentage BrdU-positive cells coexpressing PR in ducts and end buds after C, E2, P, or E2+P treatment are presented. The percentage of BrdU-positive cells in ducts increased in response to E2, P, or E2+P treatment compared with control (*P < 0.05). The percentage of BrdU-positive cells increased in end buds compared with ducts in E2, P, or E2+P (#*P *< 0.05).

To examine the sensitivity and response of the mammary gland to P before the onset of puberty, 3-week-old BALB/c mice were OVX and treated with a low dose of E2 or P [19]. Notably, the single low-dose P treatment produced EB formation similar to that observed with E2 (Figure 5A) and similar to the results with 5-day P treatment during puberty (Figure 1A). Control-treated mice had only rudimentary mammary glands with no EBs present (Figure 5A). PR expression after low-dose hormone treatment was detected at similar levels across all treatments (Figure 5B). The anti-estrogen ICI 182,780 inhibited E2-induced EB formation (Figure 6A), but not P-induced EB formation. Treatment with the anti-progestin RU486 inhibited P-induced EB formation (Figure 6B).

Similar to the 5-day treatment during puberty, BrdU uptake in epithelial cells (PR^+ ^and PR negative (PR^-^)) within EBs was significantly higher than in ducts after the single low-dose treatment and was similar among E2, P, and E2+P treatments (Figure 6C). Additionally, a substantial percentage of PR^+ ^cells in both EBs and ducts exhibited BrdU uptake (Figure 6C).

**Figure 7:**
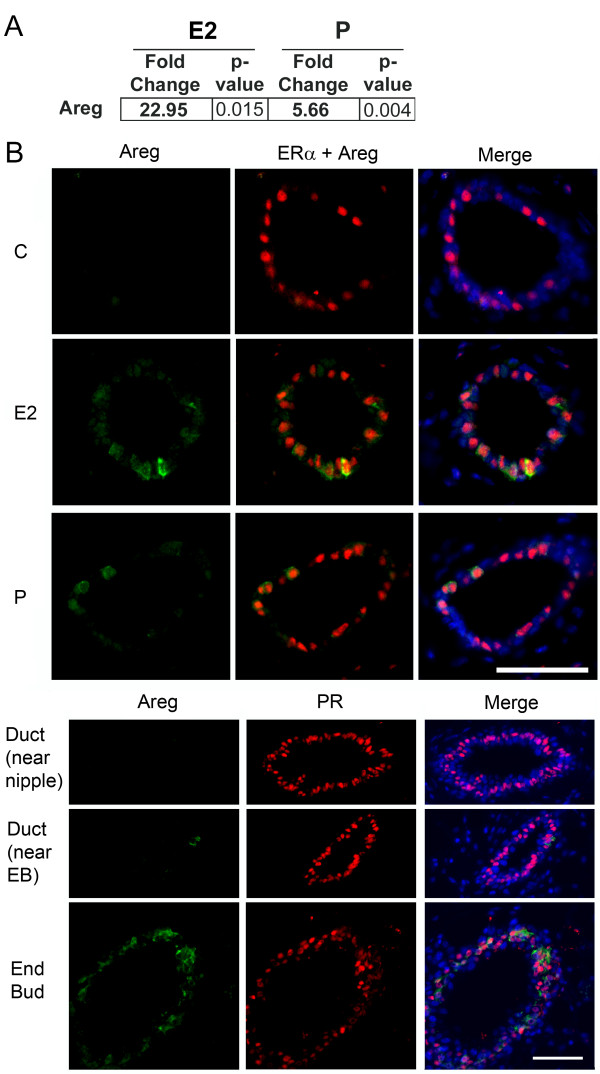
**17-β-Estradiol regulates amphiregulin, and progesterone regulates both amphiregulin and RANKL in the prepubertal mammary gland**. Prepubertal 3-week-old BALB/c mice were OVX, allowed to recover for 2 weeks, and treated once with vehicle control (C), E2, or P, as described in the Materials and Methods section. **(A) **Expression analysis of amphiregulin (AREG) by RT-PCR. Fold change is relative to control treatment. **(B) **Dual immunofluorescence detection of AREG (green) and either ERα (red, on left) or PR (red, on right). AREG expression increased in response to E2 or P in ERα-expressing cells and was most strongly expressed in end buds in response to P treatment. Nuclei were counterstained with DAPI (blue). Scale bar, 25 μm.

Analysis of *Areg*-encoding mRNA in the prepubertal mammary gland by quantitative RT-PCR showed that *Areg *expression was increased by single low-dose treatment with P (5.66 fold) or E2 (22.95 fold) compared with vehicle controls (Figure 7A). AREG protein expression, as measured with immunofluorescence, also increased in the prepubertal mammary gland in response to single low-dose of either E2 or P treatment in ERα/PR-expressing cells (Figure 7B, C) and was higher after low-dose E2 treatment than after P treatment (Figure 7B). Within the mammary epithelium, AREG was most strongly induced by P in EBs (Figure 7C, EBs) and in ducts near EBs (Figure 5C, duct near EBs). In ducts farthest from the EBs, AREG was not detected (Figure 7C, duct near nipple). This gradient of AREG expression was observed for all three hormone treatments, consistent with a preferential localization of AREG in EBs and a dominant role in EB formation.

In the contrast to the results for AREG, no treatments, including a single P injection, increased RANKL expression in the prepubertal 3-week-old BALB/c mammary gland (Additional File 1, Figure S4).

## Discussion

The present results demonstrate a novel progesterone (P)-specific effect to cause end bud (EB) formation and proliferation in the developing mammary gland both before and during puberty. This effect was primarily mediated through P-induced amphiregulin (AREG) via a progesterone receptor (PR)-mediated mechanism, and AREG expression was increased by P in both ducts and EBs. Blocking AREG action by treatment with an epidermal growth factor receptor (EGFR) inhibitor completely abrogated the effects of P on EB formation and proliferation, and significantly reduced proliferation within ducts. Thus, AREG is an essential mediator of P-induced EB formation and proliferation. To our knowledge, this is the first report to demonstrate the sensitivity of the prepubertal mammary gland to P.

Four EGF-family receptors are expressed in the mammary gland at puberty: ErbB1 (EGF receptor/HERl), ErbB2 (c-neu/HER2), ErbB3 (HER3), and ErbB4 (HER4) [30]. Members of the EGF receptor family can homo- and heterodimerize [31], with the possibility that ligands for any of these receptors may be involved in pubertal mammary gland development. In addition, there are currently 13 recognized ligands of the EGFR family: EGF, heparin-binding (HB) EGF, transforming growth factor (TGF) α, AREG, epiregulin (EREG), epigen (EPG), betacellulin (BTC), and neuregulins (NRG) 1 to 6 [32]. Of the EGFR ligands, at least seven of these ligands (EGF, HB-EGF, TGF-α, AREG, EREG, EPG, BTC, NRG-1) have been detected in the virgin mouse mammary gland [30]. Although some EGFR ligands, such as TGF-α and HB-EGF, have been detected in pubertal EBs, AREG is the most highly expressed EGFR ligand in ducts and EBs during puberty, and AREG is required for normal ductal morphogenesis [26]. AREG expression increases early in puberty, starting around 18 days old, and is strongly upregulated by estrogen (E) acting through ERα [19]. E induces EB formation and proliferation in the pubertal mammary gland through epithelial estrogen receptor α (ERα)-induced AREG via a paracrine mechanism [19,33].

Our current studies extend the importance of AREG in pubertal mammary gland development to also include P-induced AREG as a mediator of EB formation and proliferation. The ability of P acting through PR to upregulate AREG expression is consistent with previous studies in the adult mouse mammary gland [27] and mouse uterus [28]. ERα and PR generally colocalize to the same epithelial cells in the pubertal mammary gland [14], and thus AREG is produced within the same luminal epithelial cell population, whether stimulated by P or 17-β-estradiol (E2). AREG induced by either P or E2 binds to the EGFR, and EGFR inhibition abrogated the effect of AREG on EB formation and proliferation. Although the possible involvement of other EGFR ligands should be noted, it is most likely that E and P act together during normal development to produce rapid ductal development, primarily through AREG. The action of both hormones in ductal development may allow more consistent stimulation throughout the estrus cycle than would be afforded by either hormone alone. These results demonstrate that pubertal mammary EB development can be stimulated by E2 or P, and challenge the predominant understanding that all pubertal mammary development is stimulated only by E. Thus, it is important to consider that other factors/mechanisms that increase expression of AREG may stimulate EB development before and/or during puberty.

Proliferation of the epithelium in response to AREG may be through direct stimulation by AREG or through growth factors produced in the stroma [34]. P exerts its effects in the mammary gland through acting on the luminal epithelium, where PR is expressed [18,21]. However, stromal EGFR has been shown to be essential for ductal development [35], and thus epithelium-derived AREG is thought to bind to stromal EGFR to produce normal ductal development and proliferation of epithelial cells through an indirect mechanism [34]. We found that all EB proliferation and most ductal proliferation in the pubertal mammary gland was induced through AREG, and that P induced proliferation of a large population of PR^+ ^cells in EBs in the pubertal mammary gland after 5 days of treatment. A similar pattern of proliferation of PR^+ ^cells was also detected after E2 treatment. A subset of luminal epithelial cells is hormone receptor positive, expressing both ERα and PR [14]. These results suggest that either P or E-induced AREG is capable of stimulating proliferation of both hormone-receptor positive and negative cells within the mammary epithelium. The sustained proliferation of hormone receptor-positive cells during puberty contrasts with results in the adult mammary gland [36]. AREG has been shown to regulate the expansion of mouse mammary epithelial progenitor cells [37], and a subset of the hormone receptor-positive population may be an early stem/progenitor population [38]. It is conceivable that proliferation of the hormone receptor-positive putative stem/progenitor cells may provide a critical target for carcinogenesis and that AREG regulation of this proliferation could contribute to breast cancer risk associated with the pubertal window of susceptibility.

Puberty, with increased hormone production leading to EB stimulation and ductal development, has been shown to be a critical period for mammary cancer susceptibility [6-8]. E is considered to be the primary ovarian hormone involved in EB stimulation and ductal elongation during puberty, with E action mediated primarily through AREG [9]; ductal growth is delayed in PR-knockout and RU486-treated mice, showing that P is also capable of stimulating ductal growth through PR [11]. However, no mechanism for P stimulation of the pubertal mammary gland has been described. Our results have now shown that P-induced AREG causes EB stimulation in both the pubertal and the prepubertal mammary glands. Many studies have focused on the potential impact of environmental estrogens on mammary gland development and cancer progression (reviewed in [39]). Collectively, our current results and the prior literature suggest that environmental factors that influence P levels, P action, mimic P, or induce AREG can also exert significant effects on the prepubertal and pubertal mammary gland. Consistent with this, the environmental pollutant perfluorooctanoic acid (PFOA) has been shown to stimulate mammary gland development in mice through increasing serum P levels, which led to an increase in growth factors, including AREG, in the mammary gland [40]. Thus, it is important to examine factors that influence both E and P action in the pubertal mammary gland, as they may affect breast cancer risk.

Further supporting the importance of AREG in the mammary gland, we found similar proliferation and AREG expression in both pubertal BALB/c and C57BL/6 mice. Mice of these two different genetic backgrounds respond differently to P in the adult mammary gland [24], yet respond similarly to P during puberty by increasing AREG expression leading to EB formation and proliferation. In the normal rat mammary gland and in hormone-dependent rat mammary cancers, AREG has also been shown to mediate E and P signaling through EGFR [41]. In the human breast, AREG is overexpressed in most ERα-positive tumors, and expression of EGFR is associated with poor prognosis and resistance to hormone therapy [42]. Whereas the role of E acting through AREG in mammary gland development and breast cancer has been emphasized [43], the current studies implicate P as another potential mediator of AREG action. The importance of AREG in two different mouse genetic backgrounds, in both the mouse and rat, and in the human breast suggests that the AREG pathway is broadly involved in ductal development and mammary proliferation, and also in mammary cancer development.

Another downstream effector of P, receptor activator of NF-κB ligand (RANKL), was also increased by P treatment in BALB/c mice, and RANKL expression was detected primarily in ducts in the pubertal mammary gland. Inhibition of RANKL in the BALB/c mammary gland significantly reduced P-induced proliferation in ducts and to a lesser extent in EBs, but did not cause EB regression. Proliferation in the adult mammary gland is mediated by P via two distinct mechanisms; an early, direct mitogenic effect on PR^+ ^cells that is dependent on cyclin D1, followed by a more robust proliferation of PR^- ^cells through RANKL [36]. Consistent with these findings for RANKL, most adult mammary gland studies conclude that P acts through a paracrine mechanism to increase proliferation of PR^- ^cells within the epithelium [24,44-46]. It is likely that RANKL is acting as paracrine mediator of P-induced proliferation within pubertal mammary ducts, similar to its action in the adult mammary gland. In contrast to the findings for AREG, P had no effect on RANKL expression in the C57BL/6 mammary gland, where RANKL expression required both E2 and P. The lack of pubertal regulation of RANKL by P in the C57BL/6 mammary pubertal gland was consistent with our previously observed lack of P-induced RANKL in the C57BL/6 adult gland [24]. Overall, these results suggest that RANKL is involved in P-induced ductal development during puberty, but unlike AREG, RANKL is not essential for P effects on EB formation.

## Conclusions

In conclusion, these studies demonstrate that the prepubertal and pubertal glands are sensitive to P, and lend further support for the ability of P to influence mammary ductal development, even in the absence of E. Given that puberty may be an important period of cancer susceptibility, an understanding and elucidation of possible endocrine disruption by factors that influence P responses and/or AREG regulation in the prepubertal and pubertal mammary gland must be considered, in addition to factors that influence E responses. We propose that factors that influence P responsiveness and other pathways that influence AREG production, in addition to E, warrant further investigation in the prepubertal and pubertal mammary gland.

## Abbreviations

E: estrogen; E2: 17-β-estradiol; EB: end bud; EGFR: epidermal growth factor receptor; ER: estrogen receptor; P: progesterone; PR: progesterone receptor; RANK: receptor activator of NF-κB; RANKL: receptor activator of NF-κB ligand.

## Competing interests

The authors declare that they have no competing interests.

## Authors' contributions

MDA designed and carried out all aspects of the mouse experiments, designed and participated in all immunofluorescence staining, analyzed and interpreted the collective results of all the experiments, and wrote the manuscript. JRL carried out the immunofluorescence experiments. JB participated in the execution of the mouse studies. RCS participated in the design of the inhibitor studies and reviewed the manuscript. SZH conceived of the study, participated in its design and data analysis, and reviewed the manuscript for important intellectual content. All authors read and approved the final manuscript.

## Supplementary Material

Additional file 1 Hormone responses in the pubertal and prepubertal C57BL/6 mammary gland. **Figure S1. Both 17-β-estradiol and progesterone induce morphologic responses in the pubertal C57BL/6 mammary gland**. Pubertal 4-week-old C57BL/6 mice were OVX, allowed to recover for 3 weeks, and then treated for 5 days with vehicle control (C), E2, P, or E2+P, as described in the Materials and Methods section. **(A) **Morphologic response to 5-day C, E2, P, or E2+P. Scale bar, 1 mm. **(B) **Immunofluorescent detection of PR expression. The values represent the mean ± SEM PR-positive luminal cells (*n *= 3 animals per treatment). The percentage of PR-positive cells in 5-day P-treated BALB/c mice was less than control (*P < 0.05). The percentage of PR-positive cells in 5-day P-treated C57BL/6 mice was less than control (#*P *< 0.05). **Figure S2. Both 17-β-estradiol and progesterone induce similar proliferative responses in the C57BL/6 pubertal mammary gland**. Pubertal 4-week-old C57BL/6 mice were OVX, allowed to recover for 3 weeks, and then treated for 5 days with vehicle control (C), E2, P, or E2+P, as described in the Materials and Methods section. Proliferation analysis by dual immunofluorescence was detection of BrdU and PR. Total percentage of BrdU-positive cells and percentage of BrdU-positive cells co-expressing PR in ducts and end buds are presented. The percentages of BrdU-positive luminal epithelial cells in ducts in response to E2, P, or E2+P treatment are greater than control (*P < 0.05). **Figure S3. Both 17-β-estradiol and progesterone regulate amphiregulin, but co-stimulation with both 17-β-estradiol and progesterone is required to regulate RANKL in the C57BL/6 pubertal mammary gland**. Pubertal 4-week-old C57BL/6 mice were OVX, allowed to recover for 3 weeks, and then treated for 5 days with vehicle control (C), E2, P, or E2+P, as described in the Materials and Methods section. **(A) **Immunofluorescent detection of AREG (green) after E2, P, and E2+P treatment. **(B) **Immunofluorescent detection in the mammary gland with antibody against RANKL (green) in E2+P-treated C57BL/6 mammary glands. Nuclei (A, B) were counterstained with DAPI (blue). Scale bar, 25 μm. **Figure S4. Progesterone fails to induce RANKL in the prepubertal mammary gland**. Prepubertal 3-week-old BALB/c mice were OVX, allowed to recover for 2 weeks, and then treated once with P, as described in the Materials and Methods section. No RANKL (green) expression was detected by immunofluorescence. Nuclei were counterstained with DAPI (blue). Scale bar, 25 μm. Click here for file
