# Supplementary figures and images for: Amphiregulin mediates progesterone-induced mammary ductal development during puberty
Source: Breast Cancer Res. 2013 May 25;15(3):R44. doi: 10.1186/bcr3431 (PMC3738150; doi:10.1186/bcr3431)

S1

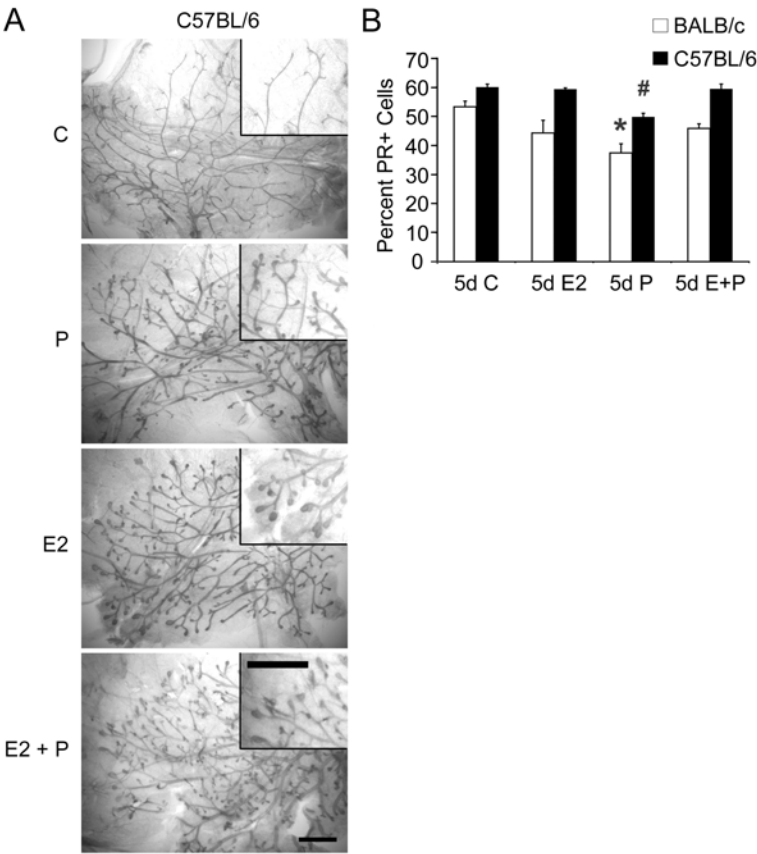

S2

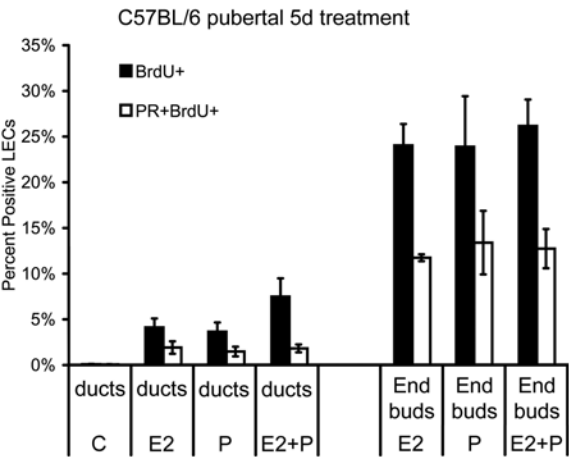

S3

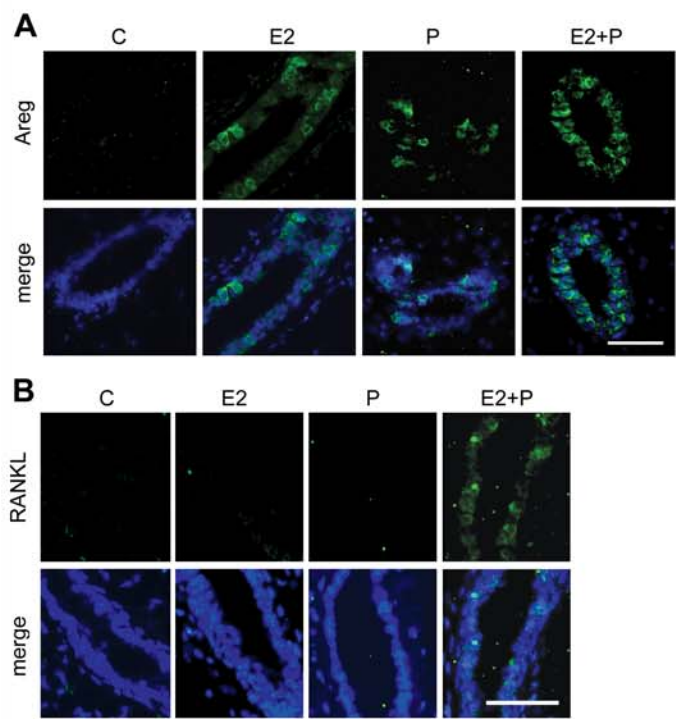

S4

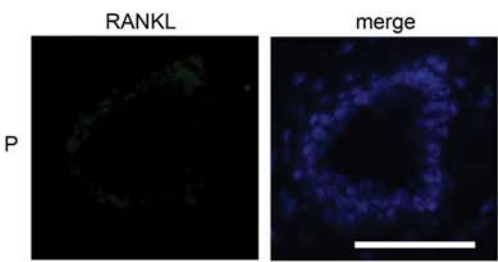

Supplement: Additional file 1 — Hormone responses in the pubertal and prepubertal C57BL/6 mammary gland. Figure S1. Both 17-β-estradiol and progesterone induce morphologic responses in the pubertal C57BL/6 mammary gland. Pubertal 4-week-old C57BL/6 mice were OVX, allowed to recover for 3 weeks, and then treated for 5 days with vehicle control (C), E2, P, or E2+P, as described in the Materials and Methods section. (A) Morphologic response to 5-day C, E2, P, or E2+P. Scale bar, 1 mm. (B) Immunofluorescent detection of PR expression. The values represent the mean ± SEM PR-positive luminal cells (n = 3 animals per treatment). The percentage of PR-positive cells in 5-day P-treated BALB/c mice was less than control (*P < 0.05). The percentage of PR-positive cells in 5-day P-treated C57BL/6 mice was less than control (#P < 0.05). Figure S2. Both 17-β-estradiol and progesterone induce similar proliferative responses in the C57BL/6 pubertal mammary gland. Pubertal 4-week-old C57BL/6 mice were OVX, allowed to recover for 3 weeks, and then treated for 5 days with vehicle control (C), E2, P, or E2+P, as described in the Materials and Methods section. Proliferation analysis by dual immunofluorescence was detection of BrdU and PR. Total percentage of BrdU-positive cells and percentage of BrdU-positive cells co-expressing PR in ducts and end buds are presented. The percentages of BrdU-positive luminal epithelial cells in ducts in response to E2, P, or E2+P treatment are greater than control (*P < 0.05). Figure S3. Both 17-β-estradiol and progesterone regulate amphiregulin, but co-stimulation with both 17-β-estradiol and progesterone is required to regulate RANKL in the C57BL/6 pubertal mammary gland. Pubertal 4-week-old C57BL/6 mice were OVX, allowed to recover for 3 weeks, and then treated for 5 days with vehicle control (C), E2, P, or E2+P, as described in the Materials and Methods section. (A) Immunofluorescent detection of AREG (green) after E2, P, and E2+P treatment. (B) Immunofluorescent detection in the m [file bcr3431-S1.pdf]
